# Mapping the distribution and tree canopy cover of *Jacaranda mimosifolia* and *Platanus* × *acerifolia* in Johannesburg’s urban forest

**DOI:** 10.1038/s41598-022-09780-y

**Published:** 2022-04-09

**Authors:** Solomon W. Newete, Khaled Abutaleb, Marcus J. Byrne

**Affiliations:** 1 Agricultural Research Council-Natural Resources and Engineering, Private Bag X79, Arcadia, Pretoria, 0001 South Africa; 2 School of Animal, Plant and Environmental Sciences, University of Witwatersrand, Private Bag X3, Johannesburg, 2050 South Africa; 3 National Authority for Remote Sensing and Space Sciences, Cairo, Egypt; 4 DST-NRF, Centre of Excellence for Invasion Biology, University of Witwatersrand, Johannesburg, South Africa

**Keywords:** Forestry, Urban ecology, Environmental sciences

## Abstract

This study investigated the distribution and the tree canopy cover (TCC) of the two most prominent street trees (*Jacaranda mimosifolia* and *Platanus* × *acerifolia*) in Johannesburg, using the multispectral SPOT 6 satellite data and field survey GPS points. The importance of the spectral bands (Blue, Green, Red and NIR) and the NDVI index in discriminating between the tree species was quantified using five separability indices (Divergence, Bhattacharyya, Transformed Divergence, Jeffries-Matusita and M-statistic). The visual comparison of the Blue band and the NDVI histograms between the two species and other vegetation type showed the lowest feature overlap, suggesting the highest separability between paired classes. This was further supported by the highest Divergence value for the Blue band (3.68) and NDVI index (2.48) followed by the M-statistic (0.8 and 0.73, respectively) indicating good to moderate separability between the two species, respectively. The results were also consistent with the RF classification where the Blue band and NDVI index were the most important variables for the discrimination between the two species with an overall accuracy of 88% (kappa = 8). The TCC of *J. mimosifolia* and *P.* × *acerifolia* constituted 38% of the total vegetation cover in the city. These findings not only would help prioritize the increase of targeted vegetation cover in low cover areas, but will also provide a valuable information for assessment and protection of vulnerable species such as *P.* × *acerifolia* from the threat of the polyphagous shot hole borer, *Euwallacea fornicatus* in Johannesburg.

## Introduction

The concept of street trees is believed to have started in mid-sixteenth century in Europe, if not earlier^1^. The formal recognition of street trees as an important component of urban planning, however, was first adopted in France during the times of Napolean Ill in 1850 by Baron Haussmann, the administrative leader of the city of Paris and its surrounding suburbs. He undertook the restructuring of the city, not only to include city parks, as was the case in London and New York, but also to make space for tree-lined boulevards and streets^2^. This desire for street trees along with parks, gardens, museums, libraries and vistas in cities was later adapted in Chicago, Sydney and across the cityscapes of Europe. The aim was to reconfigure the fragmented urban growth attributed to industrialization and to re-unite the different social classes and instil peace and order in growing cities^2^. It also spread into the rest of the world as part of various colonial expeditions from late nineteenth century as far as Spanish Manila, Dutch Batavia (now Jakarta), French Pondichéery, British Calcutta and Dutch South Africa^2^. For instance, *Cupressus sempervirens* and *Jacaranda mimosifolia* were the first two species deliberately introduced as ornamental trees to Rwanda in the first half of the twentieth century^3^. The Brazilian pepper tree (*Schinus molle*) was introduced and distributed from state owned nurseries in Australia for street trees in 1902^2^. In South Africa, the Monterey Pine (*Pinus radiata* D. Don) tree was one of the most important species introduced for timber^4^, while the common European Oak (*Quercus robur* L), London Plane (*Platanus* × *acerifolia* Willd.), *Platanus wrightii* S. Wats., of Arizona and New Mexico and the South American Jacaranda tree (*Jacaranda mimosifolia* D. Don.), were introduced by Europeans for street trees^4^. Many of these exotic ornamental species continue to dominate the urban forests and green spaces across the world as a footprint of historical colonial times. For instance, more than 70% of street trees surveyed in ten towns in the Eastern Cape Province of South Africa were aliens most of which were found in wealthy suburbs founded by European settlers^5^. Similarly, more than 75% of the ornamental trees (including street trees) of Kigali in Rwanda are exotic trees, particularly towards the inner city, as a direct outcome of the city’s colonial history^3^.

Street trees in the nineteenth century might have started as a symbol of modernity, where cities were beautifully and strategically transformed, for some political or social objectives drives in Europe^5,6^, or as shade trees to reduce heat in the colonies^2^, or for sentimental purposes planted in honour of the fallen of various wars fought. Such perceptions of street trees or urban forest has changed in our contemporary world, where the challenges of highly populated urban spaces and the rising impacts of global warming are converging as threats to urban livelihoods. Thus, street trees are not only planted for their aesthetic values, but also as the ‘lungs and kidneys’ of cities, to filter air, water and noise pollution. In addition, they reduce the urban heat island effect^7^ and decrease potential storm water flooding, and soil erosion among others. Despite the long list of ecosystem services the street trees provide, they also have a considerable social, environmental and economic disservices, which vary substantially between plant species and geographical areas^8^.

The city of Johannesburg claims to have the largest urban forest with approximately 10 million trees although this has yet to be substantiated. These are predominantly found in the wealthiest suburbs in the north of the city. Schäffler and Swilling^9^ indicated that the urban forest in the northern suburbs of Johannesburg covers 24.2% of the city’s total area as opposed to the 6.7% in the southern suburbs, where most of the historically disadvantaged citizens live. To promote species diversity and to reduce the effect of potential outbreaks of host specific pests and diseases researchers suggest a single species should be less than 10% of the street tree population^10,11^. Traditional surveys of urban trees are often tedious, time and resource demanding, and above all, constrained by lack of accessibility to private property, usually leading to deficiency of contemporary information on urban trees for city planning and development. Therefore, this study attempted to measure the distribution of two of the most common trees in the city of Johannesburg, *Jacaranda mimosifolia* and *Platanus* × *acerifolia* by mapping the green infrastructure of the city. According to Kambites and Owen^12^ this is defined as trees on streets, in gardens (private or public), in parks, along urban drainage lines and on undeveloped ridges or urban agricultural spaces.

Correctly identifying and mapping urban forest at species level using multispectral sensors with limited spectral bands is complicated by the diversity of urban trees, and overlapping canopies between different plant species. However, the continuous improvement in spatial and spectral resolution of several multispectral satellite sensors, has revitalized their importance for use in urban forest mapping. Among these is the Worldview-2, the first high-resolution satellite with eight multispectral bands (ranging from 400 to 1040 nm at 0.5–2 m intervals) first launched in October 2009. Its potential for mapping trees at species level has been proven in several studies^13^. For instance, Cho et al.^13^ used object based classification with the support vector machine (SVM) algorithm to classify three tree species and canopy gaps (bushes, bare soils, and burnt areas) in the Dukuduku Forest patch in the KwaZulu Province in South Africa, using Worldview-2 and found an overall accuracy of 89.3%. Another high-resolution multispectral sensor is, the *Systeme Probatoire d’Observation de la Terre* (SPOT-6/7) with spatial resolution of 1.5 m^14,15^. Although with only five bands, the high spatial resolution, spanning the visible and near infrared wave lengths in the electromagnetic spectrum, is suitable for urban forest mapping at species level. Lefebvre et al.^15^, using SPOT-5 (2.5 m resolution) to map tree cover in three European cities, found an overall accuracy of up to 87.5% using the SVM classification algorithm.

Despite the very obvious number of trees in the city of Johannesburg, there is little information on the tree canopy cover (TCC) of any species in the city. Thus, for the first time we investigated the TCC of two common species (Jacaranda and London Plane trees) in the streets of the city, using SPOT 6.

## Materials and methods

### Study area

**Figure 1:**
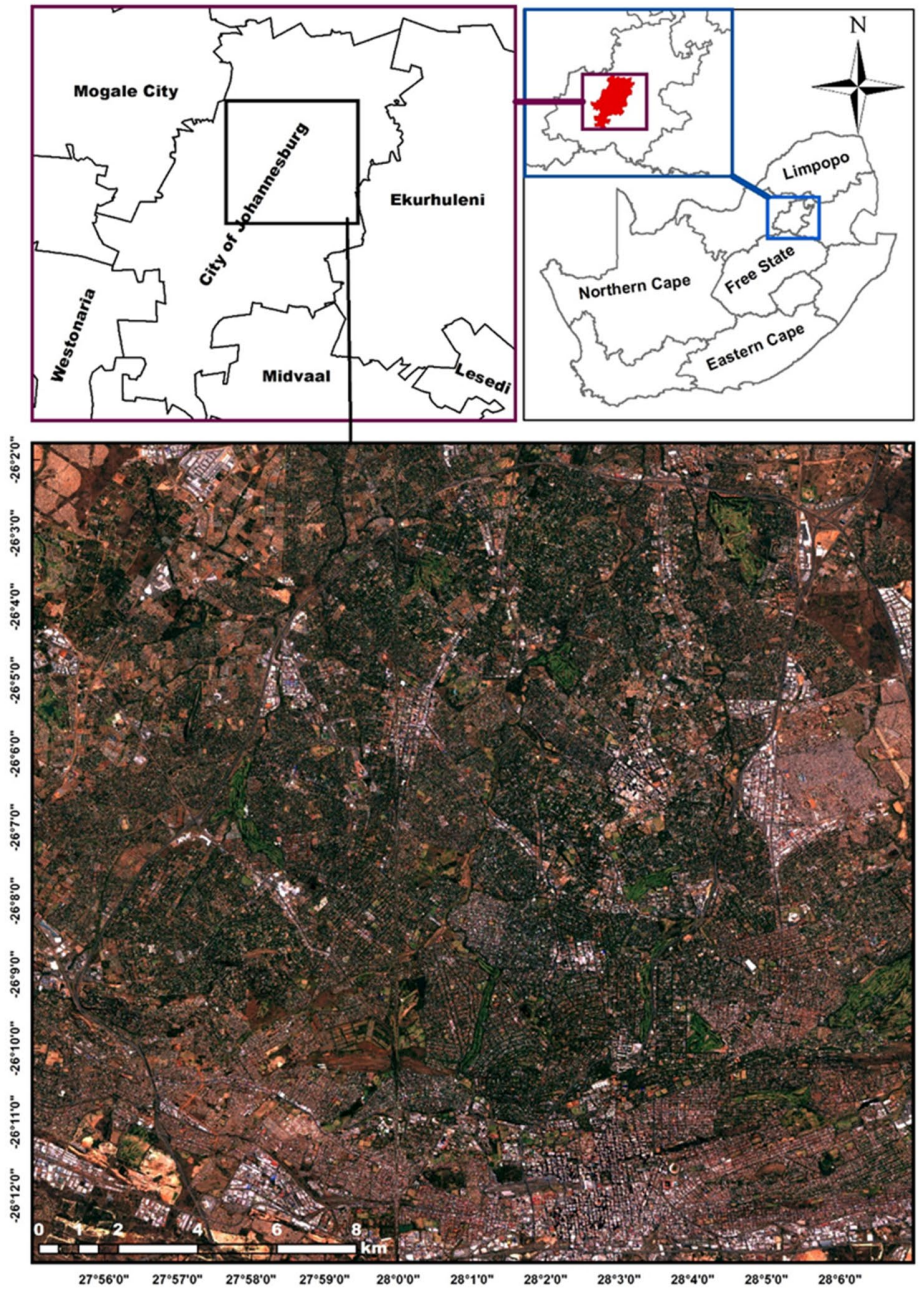
location of the study area (mainly in the north), at the city of Johannesburg, South Africa including the affluent largely white suburbs (Houghton, Rosebank, Sandton, Braamfontein) and parts of the Alexandra Township. The SPOT 6 pan-sharpened images was obtained from the South African National Space Agency (SANSA) and generated using the ArcGIS desktop software version 10.3.

This study was conducted in the city of Johannesburg (Fig. 1), the largest city in South Africa with an area of 1645 km^2^, and covers most parts of the city including the central business district (CBD), Randburg, Sandton, Houghton, Selby, Crown, and Fairland regions of the city as well as over half of the Alexandra township among others. Johannesburg is the economic and transport hub of the country situated in the province of Gauteng at an altitude of 1763 m above sea level^16^. Of the total area of the city, trees cover 16.1%, including both native and alien trees, many of which are located in the affluent largely white suburbs north of Johannesburg, planted from late nineteenth century in attempt to supress dust pollution from the mining boom that occurred around the city^9^. Thus, the green infrastructure of the city and the street trees remain unequally distributed between the privileged suburbs in the north and those marginalized by the previous apartheid regime in the south. However, this has never been properly quantified.

### Description of Jacaranda and London plane tree

*Jacaranda mimosifolia* belongs to the family of Bignoniaceae Juss^17,18^. It is originally from the Central and South America and the Caribbean. The genus has 49 species of which 39 are endemic to Brazil^19^. *Jacaranda mimosifolia* is a medium-sized (7–12 m in height) deciduous tree with a fern-like opposite bipennate leaves and terminal clusters of lavender-blue trumpet-shaped flowers^20^. London plane, *Platanus* × *acerifolia* Willd., is a deciduous tree of about 21–30 m high first found in London in 1663 as a result of interspecific hybridization between *Platanus orientalis* (Oriental plane tree) and *Platanus occidentalis* (North Eastern American plane tree)^21,22^. The genus *Plantanus* has 9–10 species and its characteristic features are fast growth, a huge crown, and smooth and exfoliating bark. It is tolerant to pruning, urban pollution and adapts to a wide range of environmental conditions^22^, making it one of the most suitable and widely used urban street trees. The leaves of London plane trees are palmate simple stalked arranged alternatively; palmately 3–7 lobbed with pseudo-palmate venation with 3–5 palmate veins^23^. Therefore, the target species have a different gross morphology, from the growth structures to their leaf shapes.

### Image acquisition and pre-processing

A SPOT 6 image consisting of four multispectral and one panchromatic bands at spatial resolutions of 6 and 1.6 m, respectively was acquired for the month of November 2017 close to the time of field date collection (GPS points) in November 2017 from the South African National Space Agency (SANSA). The multispectral image was collected in full stack and orthorectified by SANSA. Image atmospheric, radiometric and topographic corrections were done using the RStoolbox package in the R software. Atmospheric and radiometric corrections were done according to Chavez^24^ and the topographic corrections were processed according to Riano et al.^25^ using the SRTM 30 m resolution DEM and the NDVI layer computed from the image. The multispectral SPOT 6 image was enhanced to 1.5 m by fusing it with the high-resolution panchromatic band (1.5 m) using the Intensity-Hue Saturation (IHS) algorithm.

### Field survey

**Figure 2:**
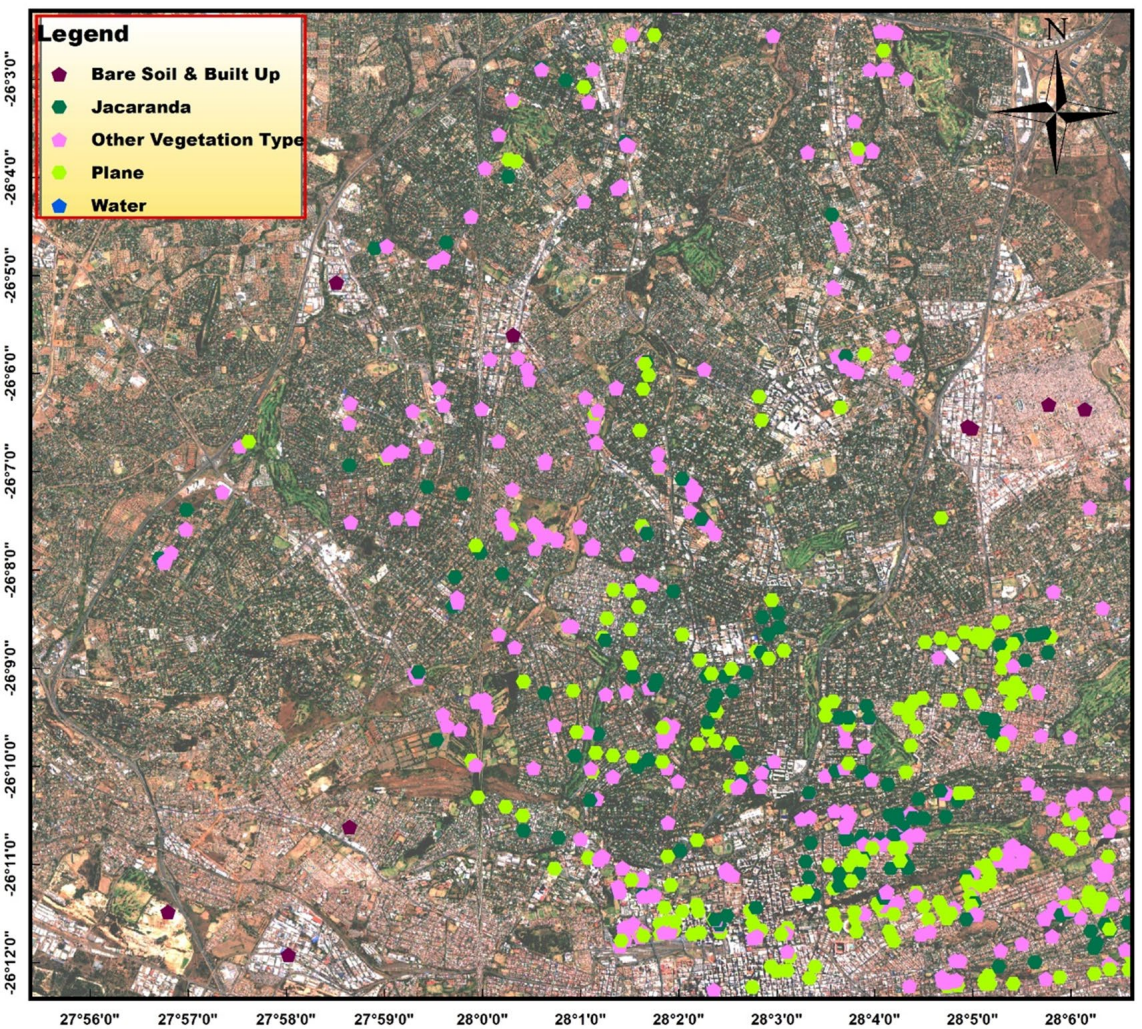
The distribution of the ground-truthing GPS points collected for mapping the distribution of two street trees, *Jacaranda mimosifolia* and *Platanus* × *acerifolia* (London Plane) and other land cover classes in the study area, the city of Johannesburg, South Africa, which includes the central business district (CBD), Randburg, Sandton, Houghton, Selby, Crown, and Fairland regions of the city as well as over half of the Alexandra township among others. The map was generated using the ArcGIS desktop software version 10.3 from SPOT 6 image obtained from the South African National Space Agency (SANSA).

A handheld Global Positioning System (GPS) receiver (Garmin eTrex 20 X) was used to collect coordinates for each of the two target street trees (*J. mimosifolia* and *P.* × *acerifolia*) and other associated vegetation types from 22 to 24 November 2017 to ground-truth the SPOT 6 satellite data. A total of 711 GPS points was collected from random street trees by visiting most of the major parks and suburbs across the study area (65 732.67 ha) and the distribution of the GPS points collected is indicated in Fig. 2. The study area was divided into suburbs and GPS points were taken selectively from every 100–200 m in the parks or by moving randomly left and right sides of the streets.

### Image processing and classification

The multispectral pan sharpened image was used to calculate the Normalized Difference Vegetation Index (NDVI)^26^, to increase the spectral capabilities of the four- band image. The calculated index was stacked with the SPOT 6 multispectral image for image classification.

#### Separability analysis

The separability between the two species (*Jacaranda mimosifolia* and *Platanus* × *acerifolia*) and other vegetation class was assessed in R “spatialEco” software version 3.4^27^ using the five most popular separability distance measures in remote sensing, namely the Divergence (D)^28^, Bhattacharyya (B)^29^, Transformed Divergence (TD)^28^, Jeffries-Matusita distance (JM)^30^ and the M-statistic (M)^31^. These tests were carried out for each of the individual bands (Blue, Green, Red and NIR) as well as for the NDVI index of the SPOT 6 image. The M-statistic measures the difference between two distributional peaks while the Bhattacharyya distance gives the similarity of two discrete or continuous distributions. The Jeffries- Matusita distance indicates the average measure between paired class densities to determine the separability quality of the classification and is robust in handling small non-parametric sample sizes^32^. Divergence is one of the most commonly used predictors of distance measures for separability of features. It is computed using mean and variance–covariance matrix of the data from which the features are selected^33^. The Bhattacharyya on the other hand, is a scalar product computed from two vectors of the features to estimate the probability of accurate classification^34^. The separability values for both D and B measures vary from 0 to *∞*^35^ and because of their non-saturating characteristic measures, the TD and J-M distance were introduced to standardize their separability range values respectively^33^. The TD values of 0 and 2 represent the minimum and maximum separability marks respectively and the value 1.5 is considered as the lower threshold above which any two classes would show a considerable spectral separability of the two features^36^. The minimum and maximum separability values for J-M distance measures ranges from 0 to 1.414^37^. The minimum threshold mark for good separability in M-statistic measurement is however, 1^38^.

#### Image classification and accuracy assessment

Random Forest (RF) is a classification algorithm that consists of ensemble decision-trees constructed using boot strapped randomly selected subset features of training samples and variables, based on which classes are assigned for the entire (unclassified) dataset^39^. The selection of a data subset which is two thirds of the original data (also known as ‘in- bag’) helps to reduce the correlation between the decision trees enhancing the level of the classification accuracy^40^. The remaining third of the data (‘out of bag’ samples) kept out of each decision tree, is used to validate the classification accuracy, of the Out of Bag (OOB) error estimate, based on which the importance of the independent variable is determined. The bigger the OOB value, the more important the variable is^39,40^. Many artificial intelligence classifiers have been proven to be of great importance for remote sensing image analysis and image classification, but what makes RF classifier superior to most of these classifiers is its ability to accommodate a large number of datasets and handle the relatively large number of variables effectively. It is also able to work with non-parametric training data, is insensitive to noise and outliers, and is robust to overfitting as more decision trees are grown^41^. This makes it more suitable for mapping tree species in more complex and heterogeneous landscapes such as an urban environment.

The two-third subset (70%) of the total ground-truthing GPS dataset was used as training data. The remaining set of the data (30%) was used for validation of the Random Forest classifier to determine how accurate the classification of the SPOT 6 satellite data was by generating overall, producer’s and user’s accuracies, and the kappa coefficient. The reference data, which represents the correct data acquired from the field survey was displayed on a confusion matrix table, and the rows that represent the classification produced from the remote sensing satellite data is displayed on the rows^42^. The overall accuracy was computed by dividing the sum of all the numbers correctly classified for each of the classes (the sum of the major diagonal values in the matrix-table) by the total number of observations (total reference number) collected from the field. On the contrary, dividing the number of correct samples in each category (column or row) by the total number of samples in the respective column or row gives ‘producer’s or ‘users accuracy’, respectively^42^.

The tree canopy cover for Jacaranda and Plane trees in each of the suburbs in the city of Johannesburg was calculated by overlaying the suburb boundary shape file obtained from the South African Global Data Base with minor modification to label some of the absent suburb names using Google Earth information.

## Results

### Separability analysis

**Figure 3:**
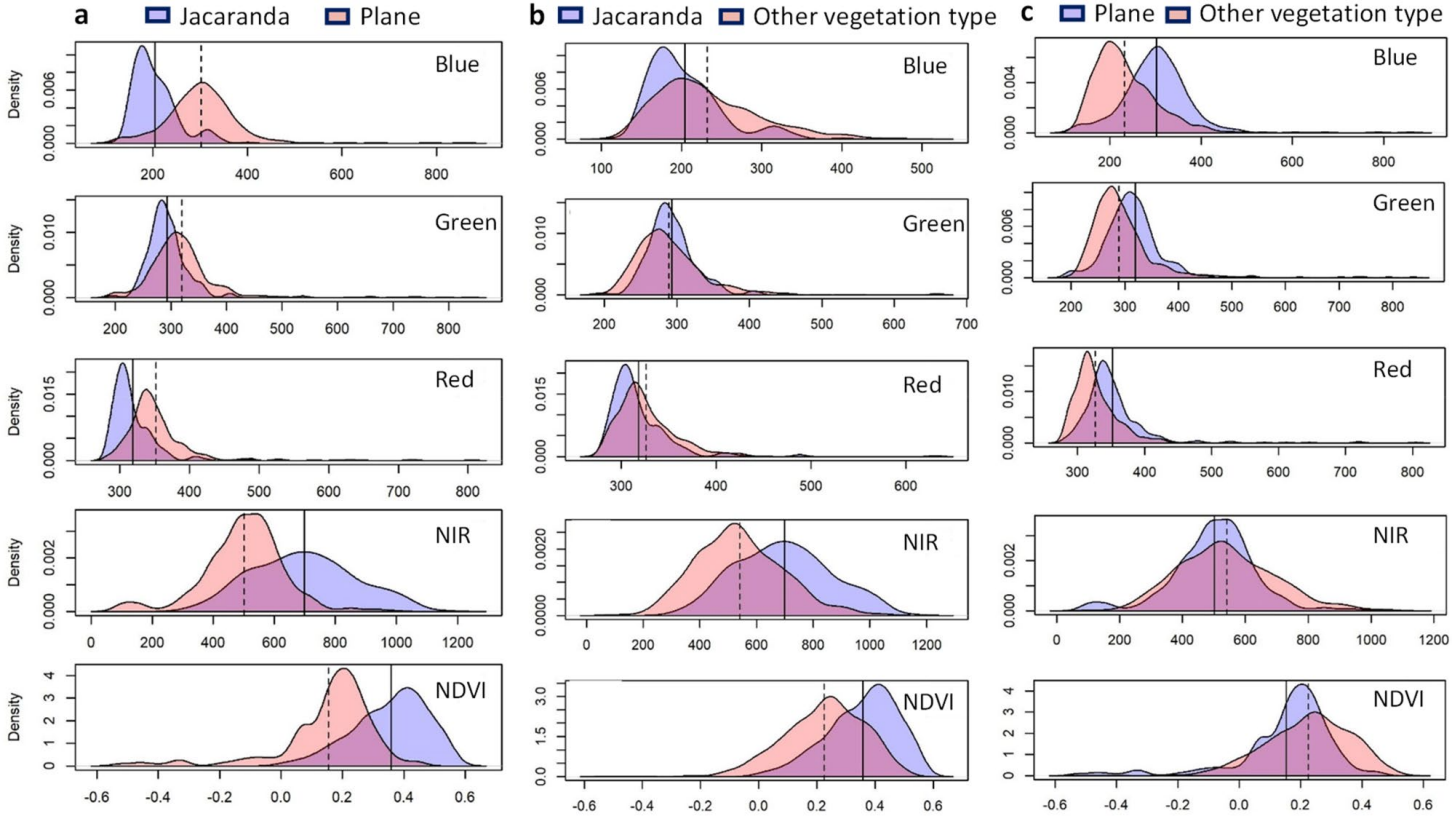
The distribution of the data for each of the paired three classes of trees and other vegetation (**a**) Jacaranda versus Plane, (**b**) Jacaranda versus other vegetation type and (**c**) plane versus other vegetation type, in the four bands (Blue, green, red and NIR) and NDVI index. Histograms with the least spectral overlap between each of the paired classes show the best separability between them. The different spectral bands and vegetative indices were generated using the ArcGIS desktop software version 10.3 from SPOT 6 pan-sharpened images obtained from the South African National Space Agency (SANSA).

The separability between the pure pixels of the two species (*Jacaranda mimosifolia,* and *Platanus* × *acerifolia*) and the class of ‘other vegetation’ was investigated using the Blue, Green, Red and NIR bands and NDVI index of SPOT 6 images. The histograms of these indices were plotted for each of the paired combinations of the three classes and the levels of the spectral confusion between the classes were visually compared to determine the ability of each indices in effectively separating between the classes. The smallest spectral overlap was observed for the Blue band histograms between the *J. mimosifolia* and *P.* × *acerifolia* followed by the NDVI and NIR indices (Fig. 3). On the other hand, the NDVI and the Blue band histograms between the paired combinations of each of the two species and other vegetation, showed the smallest spectral overlap, respectively. The visual comparisons of the Green histograms between any pair of the classes showed the biggest overlap followed by the Red band histograms, indicating the failure to discriminate between these classes. This is further supported by the results of the separability analysis in Table 1.

**Table 1:** The performance analysis of the four spectral bands (Blue, Green, Red and NIR) and NDVI index of SPOT 6 data to effectively discriminate between the pure spectral pixels of *Jacaranda mimosifolia,* and *Platanus* × *acerifolia* (London Plane) and other vegetation type using the separability measurements, Bhattacharyya distance, Jeffries-Matusita distance, M-statistic, Divergence and Transformed Divergence.

Spectral region	Paired comparison between classes		Separability indices				
	Class 1	Class 2	B	J-M	M	D	TD
Blue	Jacaranda	Plane	**0.366**	**0.613**	**0.804**	**3.677**	**0.737**
Green	Jacaranda	Plane	0.102	0.195	0.249	0.987	0.232
Red	Jacaranda	Plane	0.110	0.208	0.398	0.984	0.231
NIR	Jacaranda	Plane	0.226	0.404	**0.657**	**1.898**	0.422
NDVI	Jacaranda	Plane	0.284	0.495	**0.733**	**2.468**	0.531
Blue	Jacaranda	Other vegetation	0.059	0.114	0.255	0.510	0.123
Green	Jacaranda	Other vegetation	0.008	0.016	0.050	0.066	0.016
Red	Jacaranda	Other vegetation	0.013	0.025	0.119	0.104	0.026
NIR	Jacaranda	Other vegetation	0.122	0.230	0.492	0.985	0.232
NDVI	Jacaranda	Other vegetation	0.137	**0.256**	**0.513**	**1.125**	0.262
Blue	Plane	Other vegetation	0.132	**0.247**	**0.501**	**1.089**	0.254
Green	Plane	Other vegetation	0.072	0.139	0.271	0.645	0.155
Red	Plane	Other vegetation	0.115	0.217	0.326	**1.114**	0.260
NIR	Plane	Other vegetation	0.015	0.029	0.137	0.119	0.029
NDVI	Plane	Other vegetation	0.033	0.065	0.239	0.267	0.066

NB, numbers in bold indicate the highest separability values achieved by each of the separability indices.

The performance of the four spectral bands (Blue, Green, Red and NIR) and the NDVI index were assessed for classification of the SPOT 6 imagery using the spectral separability tests, Bhattacharyya distance (B), Jeffries-Matusita distance (JM), M-statistic (M), Divergence (D) and the Transformed Divergence (TD). Based on the respective threshold values against which good separability is marked for each of the separability measures, only the results in D showed high values for effective discrimination between any pair of the classes (Table 1). The highest Divergence value for separability was recorded between the classes *J. mimosifolia* and *P.* × *acerifolia* in the Blue band (3.68), followed by NDVI (2.48) and NIR (1.90). The D values between each of these species and the other vegetation class were, however, low with poor discrimination ability between them. These values were in the NDVI index (1.12) for the paired classes of *J. mimosifolia* and other vegetation and in the Red (1.11) and Blue (1.09) bands for the paired classes of *P.* × *acerifolia* and other vegetation (Table 1). The M-statistic also resulted in moderate separability between the two classes *J. mimosifolia* and *P.* × *acerifolia* in the Blue band (0.80) followed by the NDVI (0.73) and NIR (0.65) compared to the other separability measures B, J-M and TD, the highest values of which were only 0.37, 0.61 and 0.74, respectively for the two species.

### Assessment of variables’ importance

**Figure 4:**
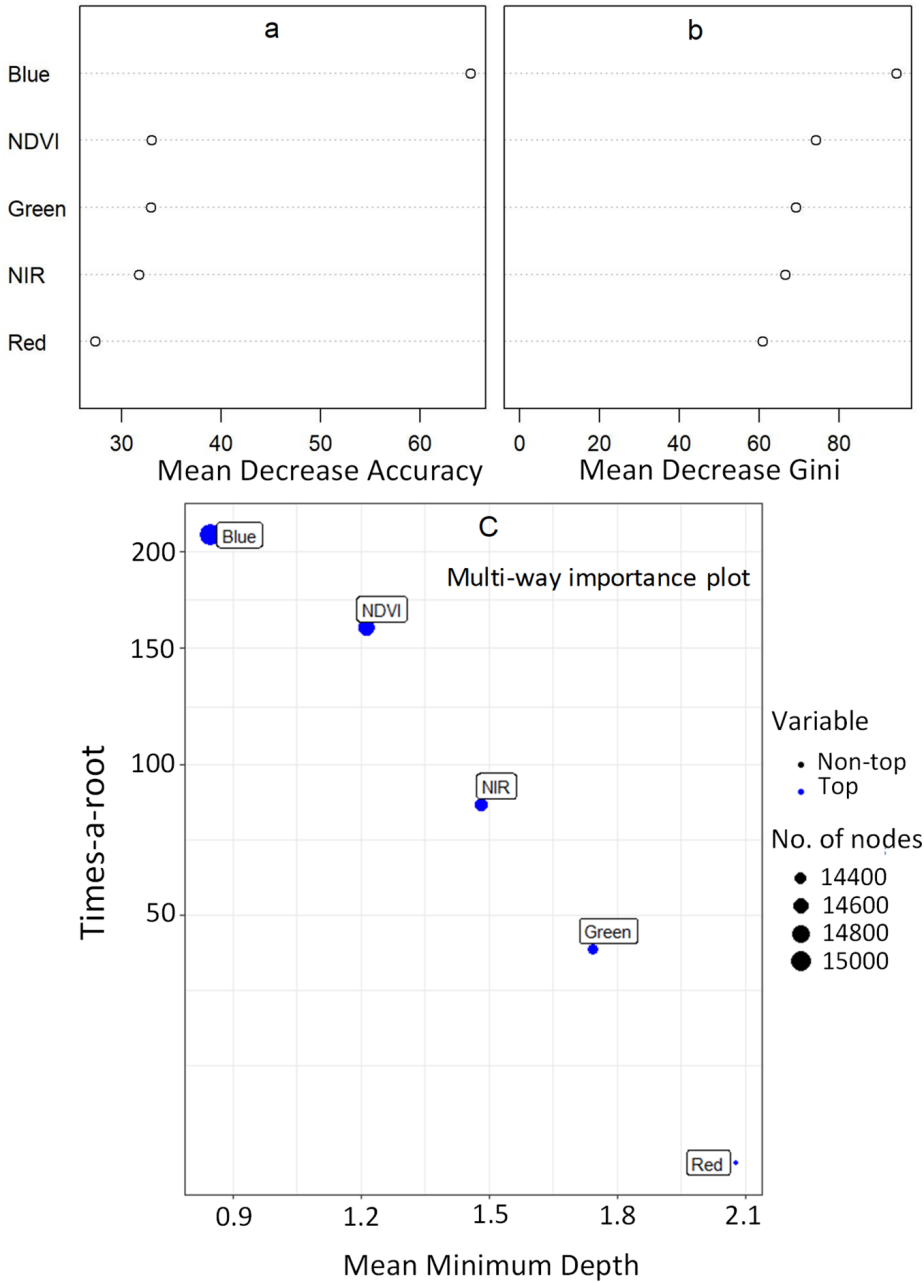
The importance of the four bands (Blue, Green, Red and NIR) and the NDVI index in the Random Forest classification of the SPOT 6 imagery (**a**) mean decrease in accuracy, (**b**) mean decrease in Gini and (**c**) multiple way importance plot showing the magnitude of contribution to RF classification between the classes *Jacaranda mimosifolia, Platanus* × *acerifolia* (London Plane) and other vegetation types in the city of Johannesburg.

The importance of each of the variables which includes the four bands (Blue, Green, Red and NIR) and the NDVI index were computed for Random Forest classification of the SPOT 6 imagery. The most important variable is indicated by the highest Mean Decrease Accuracy (MND) and Mena Decrease Gini (MDG). Thus, the Blue band had the highest contribution to the effective classification of the data set followed by the NDVI and Green band (Fig. 4a, b). This order of variables was, however, slightly different when a multiple-way importance was used to compute the contribution of each of the variables. The variables with the highest contributions were ranked in ascending order as Blue > NDVI > NIR > Green > Red (Fig. 4c).

### Accuracy assessment

The random forest (RF) classification of the SPOT 6 datasets produced an overall accuracy of 88% with kappa value of 0.8 (Table 2). Both *J. mimosifolia* and *P.* × *acerifolia* recorded 70% of producer’s accuracies. The user’s accuracy was, however, greater in the latter, with 72% and 92%, respectively. This is because there was a 17% of spectral confusion between the *J. mimosifolia* and the class designated as ‘other vegetation’ as opposed to only 4% in the *P.* × *acerifolia*.

**Table 2:** Confusion matrix obtained using the Random Forest classifier for land cover types, *Jacaranda mimosifolia, Platanus* × *acerifolia* (London Plane) and other vegetation from SPOT 6 imagery of Johannesburg, South Africa.

Classes	Random forest-confusion matric				
	*J. mimosifolia*	*P. acerifolia*	Other vegetation	Total	UA%
*J. mimosifolia*	21	3	5	29	72
*P.* × *acerifolia*	1	23	1	25	92
Other vegetation	8	7	145	160	91
Total (column)	30	33	151	214	85
PA %	70	70	96	79	88

Overall accuracy = 88%; kappa = 0.8.

UA denotes the ‘User’s accuracy and PA denotes the ‘Producer’s accuracy.

**Figure 5:**
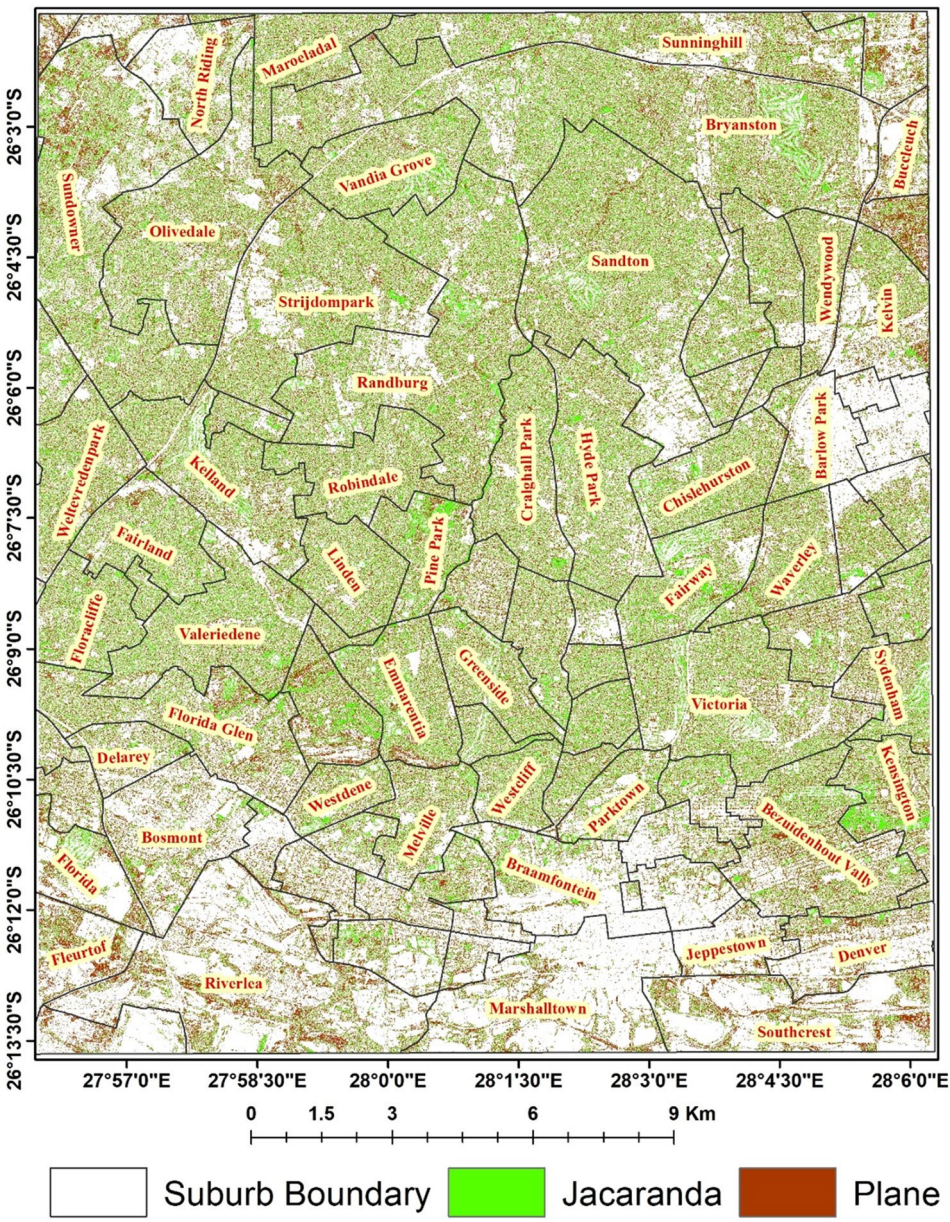
The distribution map of two common street trees (*Jacaranda mimosifolia* and *Platanus* x *acerifolia*) in the suburbs of Johannesburg City, South Africa classified using SPOT 6 data obtained from the South African National Space Agency (SANSA).

The SPOT 6 image classification using RF classifier was able to effectively map the distribution of *J. mimosifolia,* and *P.* × *acerifolia* in the city of Johannesburg with an overall accuracy of 88% and a Kappa coefficient of 0.8 (Fig. 5). The two species made up 18.4% and 19.6%, respectively of the total area under investigation (65 732.67 ha). The total vegetation cover of the study areas, which includes the two species, was 65.9% of which 38% was the total canopy cover (TCC) of *J. mimosifolia,* and *P.* × *acerifolia* (Table 3).

**Table 3:** The percentage cover of the *Jacaranda mimosifolia,* and *Platanus* × *acerifolia* (London Plane) in the city of Johannesburg, South Africa.

Class	Area (km^2^)	Percentage cover (%)
*Jacaranda mimosifolia*	72.90	18.40
*Platanus* × *acerifolia*	77.80	19.64
Other vegetation type	110.46	27.88
Other non-vegetation	135.18	34.08

The area for Jacaranda and Plane tree canopy cover for each of the suburbs in the city was estimated from the map classified using the SPOT 6 satellite imagery and Bryanston (5.3 and 5.0 km^2^), Sandton (3.9 and 3.3 km^2^), Randburg (3.7 and 3.2 km^2^), Sundowner (3.0 and 3.2 km^2^), , Bezuidenhout Valley (1.4 and 2.0 km^2^), Hyde Park (1.8 and 1.5 km^2^), Valeriedene (1.8 and 1.4 km^2^), Emmarentia (1.2 and 1.0 km^2^), Sunninghill (1.8 and 2.0 km^2^), and Craighall Park (1.3 and 1.1 km^2^), respectively, amongst the suburbs with the largest tree canopy coverage (Table 4). Some of the suburbs were partially clipped (Fig. 5 above) and therefore, the TCC area estimated for such suburbs is computed partially.

**Table 4:** Plane and jacaranda canopy tree coverage for each of the suburbs in the city of Johannesburg.

Suburb	Jacaranda (km^2^)	Plane (km^2^)	Suburb	Jacaranda (km^2^)	Plane (km^2^)	Suburb	Jacaranda (km^2^)	Plane (km^2^)
Kensington	0.911	0.846	Kelland	1.542	1.385	Floracliffe	0.962	0.738
Sydenham	0.614	0.657	Robindale	1.189	1.035	Northcliff	0.141	0.112
Victoria	2.082	2.001	Cosmo_City	0.031	0.195	Montgomery_Park	0.736	0.906
Cheltondale	0.561	0.711	Olivedale	2.143	2.358	Franklin_roosevelt_Park	0.130	0.136
Sunninghill	1.772	1.983	Randburg	3.723	3.200	Fontainebleau	0.227	0.200
Broadacres	0.072	0.238	Sandton	3.904	3.343	Vandia_Grove	1.447	1.066
Sundowner	2.978	3.255	Strijdompark	2.275	2.256	North_Riding	0.452	0.684
Bosmont	0.526	1.102	East_bank	0.187	0.577	Maraisburg	0.167	0.351
Zandspruit	0.003	0.003	Bryanston	5.345	5.047	Delarey	0.308	0.414
Maroeladal	0.898	0.868	Alexandra	0.041	0.128	Florida	0.425	0.637
Parktown_North	0.661	0.582	Marlboro_South	0.015	0.052	Coronationville	0.100	0.169
Jeppestown	0.137	0.375	Morningside_Manor	0.604	0.643	Rossmore	0.192	0.219
Noordgesig	0.069	0.318	Doornfontein	0.116	0.233	Langlaagte_North	0.131	0.352
Buccleuch	0.184	0.405	Marshalltown	0.461	1.086	Crown_North	0.079	0.173
Southcrest	0.513	1.405	Weltevreden Park	1.060	1.088	Fordsburg	0.051	0.115
Homestead_Park	0.306	0.387	Halfway_House	0.323	0.612	Mayfair	0.396	0.573
Juubert_park	0.017	0.023	Melville	0.668	0.712	Langlaagte	0.038	0.070
Braamfontein	0.465	0.559	Valeriedene	1.809	1.449	Wendywood	0.541	0.657
Berea	0.170	0.290	Forest Town	0.285	0.277	Kelvin	0.655	0.945
Denver	0.288	0.736	Fairland	1.068	0.925	Lombardy_west	0.263	0.328
Bezuidenhout_Vally	1.449	2.001	Morningside	0.625	0.583	Dorelan	0.077	0.068
Parktown	0.479	0.451	Chislehurston	1.133	0.930	Pine_Park	0.922	0.653
Riverlea	0.985	2.668	Strathavon	0.217	0.252	Saxonwold	0.446	0.344
Westdene	0.405	0.431	Simba	0.178	0.181	Rosebank	0.146	0.138
Fleurtof	0.199	0.756	Lombardy_east	0.002	0.002	Parkwood	0.263	0.214
Florida_Glen	1.325	1.659	Fairway	1.181	0.848	Parkhurst	0.332	0.461
Greenside	0.801	0.734	Waverley	0.903	0.898	Parkview	0.273	0.201
Emmarentia	1.205	1.038	Gresswold	0.374	0.372	Westcliff	0.551	0.466
Constantia_Kloof	0.004	0.005	Glenhazel	0.382	0.355	Yeoville	0.373	0.394
Hyde_Park	1.794	1.518	Randpark_Ridge	0.274	0.249	Craighall_Park	1.321	1.128
Barlow_Park	0.282	0.390	Allens_Nek	0.004	0.003	Linden	0.912	0.802

## Discussion

The separability between the two tree species and other vegetation was examined by visually comparing the histograms derived from the spectral regions (Blue, Green, Red, and NIR) and the NDVI index in addition to the most common five separability measurements and the Random Forest algorithm used for classification of the remote sensing dataset. The Blue band and the NDVI index showed the least overlap between the histograms of *J. mimosifolia* and *P.* × *acerifolia* and between any of these species and the ‘other vegetation’, suggesting the highest separability between them. This is because the degree of separability is a function of the separation of the means and the probability distribution of the paired classes^43^. The significant feature overlapping shown in the Green, and Red bands’ histograms indicate that the three classes are inseparable with these spectral regions of the electromagnetic spectrum. This was further supported by the separability measures quantified using the Divergence (D), Bhattacharyya (B), Transformed Divergence (TD), Jeffries-Matusita distance (JM), and the M-statistic (M). The highest values for separability between pure pixels of the *J. mimosifolia* and *P.* × *acerifolia* was recorded for the Blue band and NDVI index with the Divergence measurement. Although this method is one of the most widely used separability measures in remote sensing^33^ it is often considered misleading, since it often fails to correlate well with the classification accuracies^44^. For instance, despite the highest average D value recorded for selection of the best subset of four out of eight channel sets of multispectral video data for a parametric computer classification of an agricultural area, it was ranked one of the lowest in terms of classification accuracy^44^. Contrary to this, however, our results for the D distance measurement not only showed the highest separability values between the two species in the Blue (3.7) and the NDVI (2.5) indices, but also all the other four separability measures yielded the highest values for the same two indices, although most of these values were below the threshold mark for good separability (Table 1). This suggests, despite the non-saturating behaviour of the D measure, which often leads to an increase in the statistical distance between two classes beyond the upper threshold mark of 1 for good separability^45^, the results were consistent with the M-statistic measure, which produced moderate separability between the two species in the Blue band (0.8) and NDVI index (0.73). Complete separability in M-statistic is achieved at a value of 1^46^.

Although separation between the two class distributions for TD distance measure only starts above 1.5 and becomes asymptote at 2 (complete separability)^36^, the highest two separability values were also recorded in the Blue band (0.74) and the NDVI (0.53) index. The same was true for the J-M measures with values of 0.61 and 0.5, respectively, although any value below 1 suggests poor separability between the paired classes, good separability ranges from 1.38 to 1.41^37,43^. Similarly, despite the high level of feature overlapping in the Blue and NDVI histograms between the *J. mimosifolia* and the ‘other vegetation’, all four measurements produced a similar pattern of separability values following the highest values recorded for the D measurement, the only one above the good separability mark of 1^45^. The highest D separability value (1.1) between the *P.* × *acerifolia* and the ‘other vegetation’ however, was recorded in the Red band only. Despite reported limitations of the D measurement due to its non-linear relationship with classification accuracy arising from its unbound characteristics, our results for D not only were consistent with all the other four separability measurements, but also closely related to the classification accuracy results obtained using the Random Forest (RF) algorithm.

The most important variables for the RF classification of the SPOT 6 images were determined using the highest Mean Decrease Accuracy (MDA) and Mean Decrease Gini (MDG) as well as the multiple-way variable importance analysis. The results were consistent with the visual comparison of the spectral bands and the NDVI histograms between any of the paired classes as well with the results of the five separability measurements. The Blue band and the NDVI index were the highest contributors for the accurate classification and mapping of these two common street trees in Johannesburg (Fig. 4). An overall accuracy of 88% and a kappa coefficient of 0.8 was achieved. This was almost the same as what McPherson et al.^47^ found when mapping the Tree Canopy Cover (TCC) in Los Angeles, USA with an overall accuracy of 88.6% using the high-resolution QuickBird satellite imageries along with aerial photographs and GIS data. Rahimzadeh et al.^48^ also found an accuracy of 95% using SPOT 7 pixel-based classification in a forest structure study in Hyrcanian Forests, Iran. Our results were also comparable to those of Cho et al.^13^ who found an overall accuracy of 89% in classification of three tree species using the multispectral worldview-2 data. A classification map without proper assessment of the uncertainty to highlight the presence of any error estimate often leads to misinterpretation of results^49,50^. Nevertheless, in larger sampling areas the accuracy assessment of the classification still yields and checks for issues of spectral confusion and misclassification of results. For instance, a pixel-based classification of TCC in a heterogeneous urban environment using a high-resolution worldview 2/3 imageries showed only < 5.5% of difference compared to those estimated using aerial photograph interpretation in Tokyo, Japan^51^. Thus, a spectral confusion of 17% produced between the *J. mimosifolia* and the class of ‘other vegetation’ is still within the acceptable limit of accuracy, considering the high level of complex urban heterogeneity for a city believed to have over 10 million trees. It is also considered to be the densest artificial forest in the southern Hemisphere according to Johannesburg City Parks and Zoo^52^. In addition, the narrow leaf morphology of the Jacaranda is similar to many native and alien *Acacia* species in the city. Spectral measurements taken from such closely similar trees are assumed to create spectral confusion between the different tree species^53^. The separability measurements and the RF classification method effectively mapped the two individual species and their respective tree canopy cover (TCC) to a level which is acceptable in most such studies in the literature^13,54^.

The percentage cover of a tree canopy for a given area, referred as Tree Canopy Cover (TCC), is often considered as a preferred, simple, and comparable measurement for evaluation of urban forest composition and its health status^55^. Its coverage can be compared to an inventory of all urban tree species in urban environment^54^. Our results showed a total of 65.92% of the study area under vegetation cover, of which the TCC of both *J. mimosifolia* and *P.* × *acerifolia* constituted 38% with 18.4% and 19.6%, respectively (Table 3). Although our result for TCC was only for two species, it is considerably high compared to the entire tree population canopy cover of 65% reported in Portland (Oregon), 68% in Atherton (California), and 50% in Dallas (Texas)^56^. While the lower TCC threshold for a forest is still debatable and ranges from 10 to 70%, the United Nations Framework Convention on Climate Change (UNFCCC) has set a benchmark of 10–30% or more of canopy cover to define a forest^57^. In this regard, our TCC results of 38% from only two species does indeed supports that the city of Johannesburg is a man-made jungle as often indicated reportedly. The highest TCC of *J. mimosifolia* and *P.* × *acerifolia* were concentrated in the low-density most affluent residential areas of the city including the suburbs of, *inter alia*, Bryanston, Sandton, Randburg, Sundowner, Bezuidenhout Vally, Hyde Park, Valeriedene, Emmarentia, Sunninghill (1.8 and 2.0 km^2^), and Craighall Park which showed the largest areas of tree canopy cover for the Jacaranda and Plane trees. Nevertheless, not only some of the suburbs were clipped and not fully included in the map classified using SPOT 6 imageries (Fig. 5), but also the suburbs of Saxonwold, Houghton, Park View, and Parkwood are observed to have large population of the species. But because of the suburbs’ relatively smaller size, the TCC area of the two species in Table 4 is reported smaller.

This seems to be a general trend for many other big cities of the world. McPherson et al.^54^ also found that the highest TCC (31%) was found in low-density residential areas of the Los Angeles city. Similarly, Seburanga ^3^ indicated that most of the ornamental street trees are located in the affluent suburbs in the inner city of Rwanda where the Europeans first settled.

## Conclusion

The distribution and tree canopy cover (TCC) of the two most prominent street trees (*Jacaranda mimosifolia* and *Platanus* × *acerifolia*) in the city of Johannesburg were investigated using the multispectral SPOT 6 satellite data and the Random Forest classification algorithm. The separability between the species was evaluated using the spectral bands (Blue, Green, Red and NIR) and the NDVI index and quantified with five separability measurements (Divergence, Bhattacharyya, Transformed Divergence, Jeffries-Matusita and M-statistic). Despite its unbound behaviour for a standardized upper threshold mark for good separability, the Divergence measurement showed the highest values for separability distance between *J. mimosifolia* and *P.* × *acerifolia* or the other vegetation in the Blue band and the NDVI index. This was consistent with all the results of the other separability measurements, although none of them were above the lower threshold mark for good separability between the classes. It was also consistent with the Random Forest classification where the Blue band and NDVI index were the most important variables with the highest contribution for discrimination between the different species with an overall accuracy of 88%. The total vegetation cover of the study area which covers most parts of the city was 65.92% of which 38% was the TCC of the two species.

Mapping the general vegetation cover and identifying the specific distribution of the most prevalent street trees of the city will help determine the areas of priority for potential increases of vegetation cover programme in areas of high disparity, such as those with high-dense residential areas or the informal township (e.g. Alexandra) with the lowest vegetation cover. It will also be useful to assess and protect the most vulnerable street trees such as *P.* x *acerifolia* from the polyphagous shot hole borer (PSHB), *Euwallacea fornicatus* (Coleoptera: Curculeonidae: Scolytinae) which is threatening the urban forest in Johannesburg^58^.
